# Acceptability of wearable inertial sensors, completeness of data, and day-to-day variability of everyday life motor activities in children and adolescents with neuromotor impairments

**DOI:** 10.3389/fresc.2022.923328

**Published:** 2022-12-09

**Authors:** Fabian Marcel Rast, Silvia Herren, Rob Labruyère

**Affiliations:** ^1^ Swiss Children’s Rehab, University Children’s Hospital Zurich, Affoltern am Albis, Switzerland; ^2^ Children’s Research Center, University Children’s Hospital of Zurich, University of Zurich, Zurich, Switzerland; ^3^ Rehabilitation Engineering Laboratory, Department of Health Sciences and Technology, ETH Zurich, Zurich, Switzerland

**Keywords:** rehabilitation, children with disabilities, activities of daily living, accelerometry, patient compliance, reproducibility of results

## Abstract

Monitoring the patients' motor activities in a real-world setting would provide essential information on their functioning in daily life. In this study, we used wearable inertial sensors to monitor motor activities of children and adolescents with congenital and acquired brain injuries. We derived a set of clinically meaningful performance measures and addressed the following research questions: Is the target population willing to wear the sensors in their habitual environment? Which factors lead to missing data, and can we avoid them? How many measurement days are needed to obtain reliable estimates of the children's and adolescents' motor performance? The study participants wore our sensor system for seven consecutive days during waking hours. First, we derived the daily hand use of all participants, the duration of different body positions and the wheeling activity of individuals using a manual wheelchair, and walking-related measures in individuals being able to walk. Then, we analyzed the reasons for missing data and determined the reliability of the performance measures mentioned above. The large majority (41 of 43 participants) was willing to wear the sensor system for a week. However, forgetting to reattach the sensors after charging them overnight and taking them off during bathing and swimming was the main contributor to missing data. Consequently, improved battery life and waterproofness of the sensor technology are essential requirements for measurements in daily life. Besides, 5 of 11 performance measures showed significant differences between weekdays and weekend days. The reliability, measured with the intraclass correlation coefficient, ranged between 0.82 and 0.98. Seven measurement days were enough to obtain significantly higher reliability scores than the desired level of 0.8 for all but two performance measures. In children and adolescents with neuromotor impairments, we recommend monitoring everyday life motor activities on seven consecutive days. The target population accepted this measurement protocol, it covers school days and weekend days, and the number of measurement days is sufficient to obtain reliable estimates of motor performance.

## Introduction

Children and adolescents with congenital or acquired brain injuries often have difficulties in executing everyday life motor activities, such as grasping a glass of water, transferring from a wheelchair to a car seat, or walking to school. They undertake intensive rehabilitation programs as in- or out-patients with an emphasis on fostering their functional independence in these activities. To monitor the children's progress over time and evaluate the effect of therapeutic interventions, usually, motor capacity (“what a child can do”) is measured in a standardized environment at the clinic. In the habitual environment outside of the clinic, however, motor performance (“what a child does do”) becomes much more important, and it remains unclear whether children can translate their improvements during rehabilitation into everyday life (1–3). Consequently, there is a need to assess motor performance to quantify what children and adolescents do in their habitual environment.

Self-report or proxy-report measures can be used to assess motor performance. However, these tools rely on the subjective perception of these children and adolescents, or their parents, and are prone to recall and proxy bias (4). Wearable inertial sensors overcome the limitation of subjectivity by enabling objective monitoring of motor activities in real-world settings (5). However, the most commonly used outcome measure to assess performance is activity counts, which quantifies the general level of physical activity rather than the type and quality of activities performed (6). Therefore, sophisticated algorithms are needed to derive activity-specific and clinically meaningful performance measures from data of wearable sensors.

We developed such an algorithm based on the findings of two preceding studies investigating the needs of pediatric rehabilitation (7, 8). The current algorithm determines functional hand use with wrist sensors; the duration of lying, sitting, and standing positions with a trunk and a thigh sensor; the distance and speed of self-propelled wheeling periods with a wrist and a wheel sensor; the duration, distance, and speed of walking periods, and the altitude change during stair climbing periods with a single ankle sensor; and discriminates between free and assisted walking with a sensor placed on walking aids. The algorithm can be applied in a modular way. Its outcome measures and the required sensor placement are depicted in Table 1. Then, we verified the validity of this algorithm in three subsequent studies. They showed sufficient criterion validity of the hand use measures (9), good to excellent activity classification accuracy except for stair climbing (10), and accurate gait speed estimations (11).

**Table 1:** Study groups, body-worn sensor configurations, and performance measures.

Groups	Upper limb group	Wheelchair group^a^	Walking group^a^
Body-worn sensor configurations	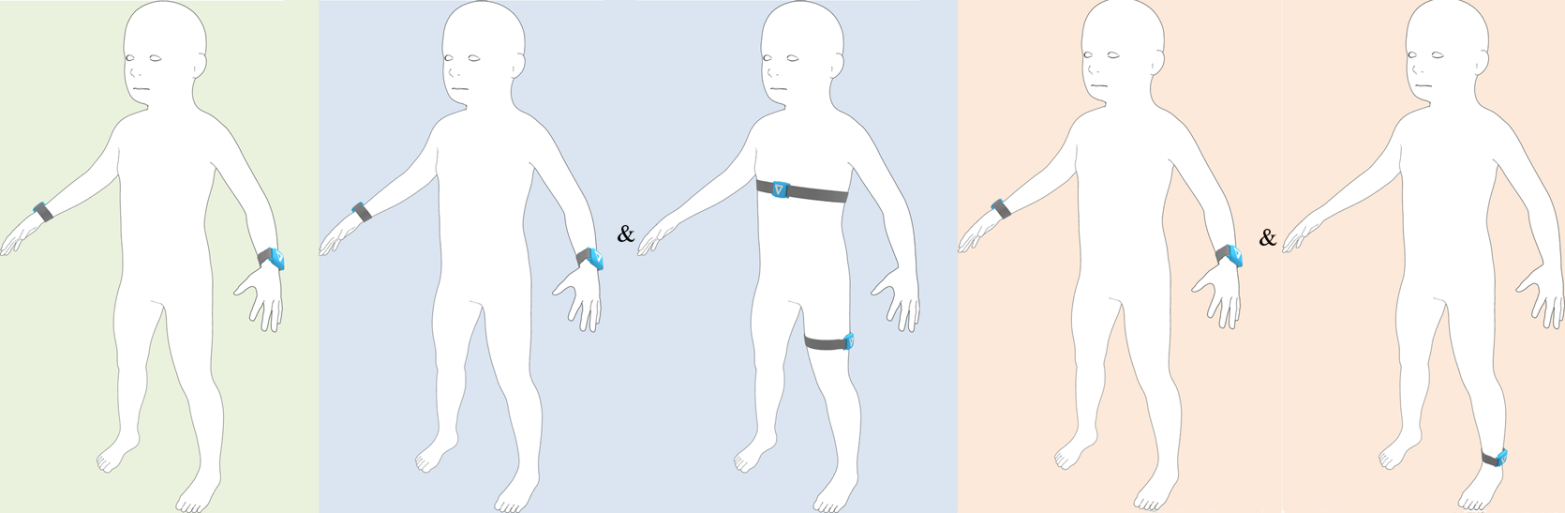
Performance measures	Hand use • Hand use (more affected) • Hand use (less affected) • Use ratio	Body positions • Time spent in lying position • Time spent in sitting position • Time spent in standing position Wheeling activity • Active wheeling distance • Active wheeling speed • Active/total wheeling distance	Walking and stair climbing • Walking duration • Assisted/free walking duration • Walking distance • Average walking speed • Going upstairs • Going downstairs

^a^ Besides body-worn sensors, we fixated additional sensors on the spokes of the wheelchair in the wheelchair group, and on walking aids in the walking group.

These validity studies were conducted in supervised experiments at the clinic to allow for the inclusion of criterion measures. However, in real-world settings, other factors such as the acceptance to wear the sensors, the completeness of data, and the naturally occurring day-to-day variability of motor activities must be considered. On the one hand, incomplete datasets could occur due to non-wearing time or technical issues of the sensor system leading to missing or biased estimates of the users' daily motor activities (12). On the other hand, a sufficient number of repeated measurement days is needed to capture the day-to-day variability of motor activities and obtain reliable estimates of the patients' overall activity levels. The literature suggests measuring performance over a week to incorporate variability between weekdays and differences between weekdays and weekends (13–15). However, this has to be reevaluated in our newly developed performance measures. Moreover, the recommendation on how many measurement days are needed will depend not only on maximizing the reliability of the performance measures but also on the children's and adolescents' willingness to wear the sensors and minimizing the burden to their everyday lives.

Therefore, we aimed to determine our sensor system's acceptability, the completeness of data, and the performance measure's reliability in a real-word setting. Children and adolescents with neuromotor impairments wore our sensor system for seven consecutive days in their habitual environment, including school days and weekend days, and we addressed the following research questions: Are children and adolescents with neuromotor impairments willing to wear the sensors in daily life? Are there other issues leading to missing data or data with insufficient quality? How many measurement days are needed to derive reliable estimates of motor performance?

## Materials and methods

This study was part of a larger ongoing study investigating the influence of contextual factors on translating rehabilitation progress into daily life. The local ethics committee approved the study protocol (BASEC-No.: 2020-00724).

### Participants

We recruited school-aged children and adolescents with congenital or acquired injuries or illnesses of the central or peripheral nervous system. They fulfilled the following inclusion criteria: ability to wheel or walk for household distances; ability to transfer between a wheelchair and a chair over a standing position in individuals who used a wheelchair; living with the mother, father, or psychological parent during the whole measurement period; no wounds or other medical conditions that prevented sensor placement; cognitive abilities to understand and follow basic verbal instructions; and signed consent form.

The participants were allocated to two of three subgroups to minimize the number of body-worn sensors and derive clinically meaningful performance measures. All participants were part of the upper limb group in which we measured their daily hand use with wrist-worn sensors. Additionally, they were allocated either to the wheelchair group or the walking group based on their primary mobility at home. In the wheelchair group, we measured the duration they spent in different body positions and their wheeling activities with additional sensors on the trunk and the thigh, while walking-related performance measures were determined in the walking group with an additional sensor on the ankle. The three subgroups and the corresponding body-worn sensor configurations and performance measures are illustrated in Table 1.

### Equipment and procedure

The study procedure comprised three parts. First, we determined the participants' motor abilities with motor assessments at the clinic to describe the study population's levels of motor impairment. Second, we monitored their motor performance with wearable inertial sensors for seven days. Afterwards, we asked the participants to rate the obtrusiveness of the sensor system. In in-patients, we conducted the motor assessments during the last week of their stay at the clinic and measured their motor performance two to four weeks after rehabilitation. We chose this time interval to allow for a habituation phase at home after the in-patient rehabilitation. In out-patients, the measurement of motor performance started directly after the motor assessments.

The motor assessments included the Melbourne Assessment 2 (MA2) to measure the quality of upper limb movements (16), and the Gross Motor Function Measure (GMFM) to determine the capacity of gross motor activities (17). Here, we performed dimensions B (sitting) and D (standing) in the wheelchair group and dimensions D and E (walking, running, and jumping) in the walking group.

To measure performance, participants were equipped with multiple ZurichMOVE sensor modules containing an accelerometer, a gyroscope, and an altimeter (18). One sensor weighs 18 g and its dimensions are 35 × 35 × 12 mm. The sensor's internal storage capacity and battery life allow for continuous recordings of roughly 72 h at a sampling frequency of 50 Hz. One of the authors demonstrated the placement of the sensors with corresponding hook-and-loop straps on the first day and provided accompanying instructions on usage and charging of the devices. During the measurement period, the participants handled the sensors themselves and were assisted by their parents when needed. All participants wore a sensor on each wrist. Those of the wheelchair group wore additional sensors on the trunk and the thigh, and we fixated a sensor on the spokes of their wheelchair. The straps of the trunk and the thigh had a silicone strip on the inside to prevent them from slipping down. Participants of the walking group wore a sensor on the ankle of their less-affected leg, and, if applicable, we fixated sensors on their walking aids. We instructed the participants to wear the sensors during the day and charge them overnight on a corresponding docking station. Besides, they needed to take off the sensors during bathing and swimming activities. They received a leaflet and instruction videos to ensure the proper replacement of the sensors (19, 20). Moreover, they were encouraged to journalize each non-wearing period. After seven days, the participants' parents were asked how obtrusive it was for their child to wear the sensors. They rated the obtrusiveness together with their child and had the choice between not obtrusive, little obtrusive, or very obtrusive.

### Data analysis

We removed non-wearing periods based on the participants' journals and visual inspections of the sensor data. Measurement days *with* non-wearing periods resulting in less than ten hours of data were considered invalid and were not analyzed (21). Saturdays and Sundays *without* non-wearing periods resulting in less than ten hours of data were kept since we assumed the participants were sleeping in. We summarized the numbers and reasons for missing or invalid measurement days. These reasons were divided into (a) concerning all sensors at once or (b) concerning a single sensor only. Eventually, we determined the performance measures for each valid measurement day as follows:

#### Upper limb group

The functional hand uses of the more and less affected sides were estimated with *functional activity counts*. Conventional activity counts were determined per second (22) but limited to periods with functional forearm elevations to minimize bias from walking and wheeling activities (9). These counts were summed to derive the daily hand use, and the use ratio was calculated by dividing the counts of the more affected hand by the counts of the less affected hand.

#### Wheelchair group

First, lying, sitting, and standing positions were classified with the orientation of the thigh and trunk sensors (10). Then, we derived the time spent in each position per day based on these classifications.

Wheeling periods were detected with the sensor on the wheel and by applying predefined rules to the gyroscope data of the wheel sensor (23). Subsequently, these wheeling periods were classified as active or passive wheeling with the orientation of the wrist sensor of the dominant hand (10). First, the wheeling speed was determined by multiplying the angular rate with the radius of the wheel. Then, active wheeling speed and distance were calculated by averaging and integrating the wheeling speed during all active wheeling periods, respectively. Moreover, the ratio between active wheeling and total wheeling distance was determined.

#### Walking group

Specific characteristics of the ankle's gyroscope signal were used to identify walking periods (10). These periods were further classified as level walking or stair climbing based on the altimeter of the ankle sensor (10). In level walking periods, the walking speed and distance were determined by segmenting the data into individual gait cycles and deriving each stride length and stride time (11, 24). Besides, level walking periods were separated into free and assisted walking based on the walking aid's acceleration signal (10). Eventually, we determined the duration, distance, and mean speed of all level walking periods, the ratio between assisted and total walking duration, and the altitude change while going up- and downstairs.

### Statistical analysis of the day-to-day variability

We fitted a linear mixed-effects model to each performance measure Y using maximum likelihood:

$$Y_{ij}=\mu +\alpha_{i}+\beta_{j}+\;\varepsilon_{ij},$$

where $\alpha_{i}$ is the fixed effect of weekday i and $\beta_{j}$ is the random effect of participant j. We assumed that the random effects and the residuals are normally distributed as

$$\beta_{j}\;i.i.d.\;∼\;N\left(0,\sigma_{\;\mathrm{participants}}^{2}\right),\;\;\varepsilon_{ij}\;i.i.d.∼\;N\left(0,\sigma_{\mathrm{residuals}}^{2}\right).$$

Mixed models were chosen, since they allow for the inclusion of incomplete datasets. Participants without any valid measurement day were excluded from this analysis. Then, we conducted an F-test with the fixed effects to determine whether the performance measures differed significantly between weekdays. Post-hoc analyses were done by pairwise comparisons between the estimated marginal means of each weekday, and *p*-values were adjusted for multiple comparisons with Tukey's method. Afterward, we calculated relative reliability with the intra-class correlation coefficient (ICC) as follows (25):

$$\mathrm{ICC}\left(3,k\right)=\frac{\sigma_{\;\mathrm{participants}}^{2}}{\sigma_{\;\mathrm{participants}}^{2}+(\sigma_{\mathrm{residuals}}^{2}/k)}$$

where *k* is the number of measurement days. Initially, we set *k = 7* to reflect an ICC of the average performance measure of seven measurement days. Confidence intervals of the ICC scores were estimated with bootstrapping. Then, we determined the minimum number of required measurement days $k_{\mathrm{ICC}>0.8}$ to obtain an ICC score of which the confidence interval is above 0.8. This value reflects acceptable reliability (26). Finally, we determined absolute reliability with the smallest detectable change (SDC) (27):

$$\mathrm{SDC}\;=\;1.96*\sqrt{2}*\frac{\sigma_{\mathrm{residuals}}}{\sqrt{7}}$$

For interpretability, SDC can be expressed as a percentage value, the SDC%, which was defined as follows (28):

$$\mathrm{SDC}\%=\frac{\mathrm{SDC}}{\mathrm{grand}\;\mathrm{mean}}*100.$$

To estimate the sample size, we used the method from Walter et al. (29) With an expected ICC score of 0.9, we would need 24 participants in each subgroup to reject the null hypothesis (ICC = 0.8) with a Type I error of 5%.

We determined the performance measures in MATLAB R2018b (MathWorks, Natick, Massachusetts, USA) and conducted the statistical analysis in R 4.1.2. (R Core Team, Vienna, Austria).

## Results

The upper limb group consisted of 43 participants. Eleven of those used a manual wheelchair (wheelchair group), and 31 walked for household distances (walking group). One participant was only recruited for the upper limb group. In the walking group, six participants used a walker, two used crutches, and two used both devices in daily life. The remaining 21 participants walked freely. The participants' demographics, motor abilities, and obtrusiveness ratings are listed in Table 2.

**Table 2:** Participants’ demographics, motor abilities and obtrusiveness rating.

	Upper limb group	Wheelchair group	Walking group
Demographics			
Sample size	43	11	31
Gender (female/male)	15/28	4/7	11/20
Age (years)	11.9 [8.8,13.7]	11.7 [9.2,13.0]	11.9 [8.7,14.0]
Di agnoses (cerebral palsy/acquired brain injury/spina bifida/other)	21/10/6/6^a^	9/2/0/0	11/8/6/6^a^
Motor assessments			
MA2 more affected side (%)	67.4 [50.6,92.1]	NA	NA
MA2 less affected side (%)	89.1 [76.6,93.5]	NA	NA
GMFM-B (%)	NA	58.3 [37.1,74.2]	NA
GMFM-D (%)	NA	7.7 [5.1,15.4]	82.1 [67.9,94.9]
GMFM-E (%)	NA	NA	75.0 [40.6,94.1]
Questionnaire			
Obtrusiveness (not/little/very/missing)	25/14/1/3	8/2/1/0	16/12/0/3

The numbers are counts or medians [25th, 75th percentile].

MA2, Melbourne Assessment 2; GMFM, Gross motor function measure, B, sitting, D, standing, E, walking, running & jumping.

^a^ Hereditary neuropathy (2), congenital malformation of the brain (2), brain atrophy (1), paralytic gait (1).

Most participants rated wearing the sensors as not obtrusive and all but one as not or little obtrusive. Besides, 14% of the measurement days were missing or invalid because of reasons concerning all the sensors at once. Additionally, specific performance measures could not be determined because the data of single sensors were missing. The rate of missing measurement days depended on the sensor position and ranged between 0% and 26%. Taking reasons concerning all sensors and single sensors together, the rate of missing values ranged between 11% and 36%. Specific numbers and reasons for missing values are shown in Table 3. Forgetting to put on the sensors in the morning, forgetting to reattach them after showering or bathing, and prolonged swimming activities resulted in 20 invalid measurement days with less than ten hours of data. Two children refused to wear the sensors after one and two days, respectively, resulting in 11 missing measurement days. Families often forgot to charge the sensors on assistive devices or did not replace them in the morning, which resulted in 20 missing measurement days. The sensor on the thigh slipped down to the shank on 9 measurement days, resulting in confusion between sitting and standing positions and thus in invalid datasets.

**Table 3:** Numbers and reasons for missing measurement days divided into concerning all sensors at once or a single sensor only.

Reasons concerning all sensors		Reasons concerning single sensors	Sensor position					
			Wrist	Trunk	Thigh	Wheel	Ankle	Aid
Not willing to wear the sensors	11	Not worn/fixated	1			7	1	4
Invalid: wearing time <10 h	20	Forgot to charge				7		2
Sickness/injury/quarantine	8	Technical issue	5			6	9	
Holiday	3	Sensor slipped down			9			
Sum of missing measurement days	42	Sum of missing measurement days	6	0	9	20	10	6
Total number of measurement days	301	Total number of measurement days	301	77	77	77	217	70
Missing measurement days [%]	14%	Missing measurement days [%]	2%	0%	12%	26%	5%	9%

The results of the day-to-day variability, including the F-test, the ICC, and the SDC are shown in Table 4. Descriptive statistics of the wearing time, the performance measures, and the pairwise comparison between weekdays are illustrated in Supplementary file S1. Participants without valid measurement days were excluded from this analysis explaining the altered number of participants and missing values in Table 4 compared to Tables 2, 3. One participant had to be excluded from the upper limb group, one participant from the wheelchair group, while two participants had to be excluded from the walking group. Five performance measures showed significant differences between weekdays. Participants were less active on Saturday and Sunday than on school days. This trend was observed in all performance measures related to the duration or amount of a motor activity except for stair climbing. The measurement of speed and ratios did not significantly differ between weekdays. The ICC (3,7) ranged between 0.82 and 0.98. Upper limb-, standing-, and walking-related performance measures revealed higher ICC scores and would require one to two measurement days to obtain reliable outcomes. The remaining performance measures would require 5–8 measurement days. The SDC% ranged between 16% and 98%, with lower values for upper limb and speed-related measures, and for the duration of sitting and walking activities.

**Table 4:** Day-to-day variability of each performance measure.

Performance measures	Descriptive statistics			Fixed effects		Random effects		Intra-class correlation (ICC)			Smallest detectable change (SDC)	
	n_participants_	missing	mean ± SD	F-statistic	*p*-value	*σ*_participants_	*σ*_residuals_	ICC (3,7)	95%–CI	k_ICC >0.8_	SDC	SDC[%]
Hand use (more affected) [kilo counts]	42	14%	1673 ± 7420	4.64	0.00	682	277	0.98	0.98–0.99	1	291 kilo counts	17%
Hand use (less affected) [kilo counts]	42	14%	2247 ± 8850	7.26	0.00	825	334	.98	0.97–0.99	1	349 kilo counts	16%
Use ratio [%]	42	14%	76 ± 20	0.51	0.80	56	8	0.98	0.98–0.99	1	8%	NA
Duration in lying position [min]	10	20%	115 ± 121	0.88	0.51	90	81	0.90	0.87–0.98	5	85 min	74%
Duration in sitting position [min]	10	20%	531 ± 147	1.96	0.09	108	98	0.89	0.85–0.98	5	103 min	19%
Duration in standing position [min]	10	20%	55 ± 66	3.14	0.01	54	31	0.95	0.94–0.99	2	33 min	60%
Active wheeling distance [m]	10	36%	830 ± 715	2.66	0.03	516	485	0.89	0.83–0.98	5	508 m	61%
Active wheeling speed [m/s]	10	36%	0.32 ± 0.11	0.30	0.93	0.08	0.09	0.85	0.83–0.98	7	0.09 m/s	28%
Active/total wheeling distance [%]	10	36%	50 ± 22	0.86	0.53	18	17	0.89	0.76–0.99	8	17%	NA
Walking duration [min]	29	11%	59 ± 39	4.27	0.00	35	19	0.96	0.95–0.98	2	20 min	34%
Assisted/total walking duration [%]	9	14%	42 ± 33	0.93	0.48	25	23	0.91	0.80–0.98	5	24%	NA
Walking distance [m]	29	11%	1328 ± 1245	2.00	0.07	1075	653	0.95	0.95–0.98	2	685 m	52%
Average walking speed [m/s]	29	13%	0.72 ± 0.22	1.29	0.27	0.20	0.09	0.97	0.97–0.98	2	0.10 m/s	14%
Going upstairs [m]	26	13%	21 ± 26	0.68	0.67	16	20	0.82	0.79–0.95	8	21 m	98%
Going downstairs [m]	26	13%	−19 ± −22	0.80	0.57	15	17	0.84	0.81–0.94	7	18 m	96%

n_participants_, number of participants; SD standard deviation; CI confidence interval; k_ICC > 0.8_ number of required measurement days to obtain an ICC score of which the confidence interval is above 0.8.

## Discussion

This study investigated the acceptability of wearable inertial sensors to monitor everyday life motor activities, the completeness of data, and the reliability of motor performance measures in children and adolescents with neuromotor impairments.

### Acceptability and completeness of data

The large majority of children and adolescents was willing to wear the sensors for a week and perceived them as only minimally affecting their everyday life motor activities. This is in line with previous studies investigating the acceptability of wrist-worn sensors in typically developing children (30), and a waist-worn sensor in children with cerebral palsy (31). Refusing to wear the sensors resulted only in 4% of missing values in our study. However, there were other issues leading to missing or invalid measurement days. Insufficient wearing time, loose thigh sensors, and issues related to charging and replacing the sensors were the main reasons for missing measurement days. Our sensors' waterproofness allowed for short showers but not for bathing and swimming activities. The latter resulted in prolonged non-wearing periods, especially because of forgetting to reattach the sensors afterwards. Hence, improving the sensors' waterproofness would decrease the rate of invalid measurement days. Still, non-wearing periods can probably not be prevented completely, and future studies should implement strategies to impute missing sensor data (12). The thigh sensor could be firmly attached with adhesive tape, but studies using this approach had similar rates of missing values (32, 33). The battery life of wearable sensors needs to be improved to avoid having to charge the sensors overnight and allow 24 h-measurements over a week. Our algorithm relies on gyroscope data and Bluetooth communication between sensors to derive valid estimates of motor performance. However, these technologies have a high energy consumption and currently prevent longer measurements than two to three days.

### Day-to-day variability of performance measures

Children and adolescents were less active on weekends compared to school days. This confirms the findings of comparable studies (34–36), and underpins the need to measure performance on weekend days and weekdays to capture the children's and adolescents' motor activities comprehensively (37).

The ICC and SDC values of our study correspond to average performance measures of seven repeated measurement days, which allows for dividing the variance of the residuals by seven (see Chapter 2.4). With this, all ICC scores exceeded the desired value of 0.8, indicating sufficient reliability to discriminate patients with different levels of motor performance. However, the SDC values seem to be rather large, implying a large between-day variability of motor performance. Consequently, we recommend measuring performance over a week, even though the ICC scores of some performance measures suggest that fewer measurement days would be sufficient. The heterogeneous study population with various diagnoses and different levels of motor impairment might have led to a large between-subject variability which explains the high ICC scores despite day-to-day variability of motor performance.

Hand use and walking-related measures revealed higher relative and absolute reliability than the wheelchair group and stair climbing measures. The SDC% of the different body positions' durations were comparable to previous findings in children with cerebral palsy (34). We suggest measuring body positions with a 24 h protocol to improve reliability. Otherwise, the lying and sitting durations depend more on the waking and non-wearing time than on the patients' actual performance, which could explain the large day-to-day variability in these measures. The wheeling-related performance measures were less reliable than in adults, for whom only four measurement days are sufficient to obtain reliable outcomes (38). An explanation could be the smaller between-participant variability of wheeling activity in children compared to adults, which leads to smaller ICC scores. Thus, more measurement days are needed to obtain reliable outcomes in children, which underpins the need to measure performance over a week in children and adolescents with neuromotor impairments. In contrast, walking-related measures were more reliable than previous findings in children with cerebral palsy (34, 39). However, the comparability is limited due to differences in the study population and the number of measurement days. The altitude change during stair climbing periods revealed the lowest reliability coefficients in this study. An explanation could be the limited accuracy to detect stair climbing periods (10), which adds a source of error to the day-to-day variability.

### Study limitations

This study has three main limitations. First, the sample sizes of the wheelchair group and ambulatory children using walking aids were smaller than the desired value of the sample size calculation. This explains the larger confidence intervals in these groups, and the estimated numbers of required measurement days to obtain reliable outcomes might be too high. Still, the null hypothesis was rejected in six of the seven corresponding performance measures, indicating sufficient power in these subgroups. Another limitation could be the heterogeneity of the study population. The findings of this study might depend on age or the underlying diagnoses. However, this has to be shown in larger studies with sufficient participants in each subgroup. Last, the variability between days is composed of actual differences in movement behavior and measurement error of the sensors and the algorithm. These sources of variability cannot be disentangled with the chosen study design. Therefore, improvements in sensor technology and the underlying algorithm might enhance the reliability of performance measures. However, this needs to be investigated in future studies.

## Conclusion

In children and adolescents with neuromotor impairments, we recommend monitoring everyday life motor activities on seven consecutive days. The target population accepted this measurement protocol, it covers school days and weekend days, and the number of measurement days is sufficient to obtain reliable estimates of motor performance. However, the battery life of the chosen sensor technology should last for a week, too. This would decrease the non-wearing time during waking hours, in which the sensors ran out of battery or the users forgot to reattach the sensors after charging them. Moreover, a sufficiently long battery life would allow for 24 h-measurements and a comprehensive view of the patients' daily activities.

## Data Availability

The raw data supporting the conclusions of this article will be made available by the authors, without undue reservation.
