# Supplementary material for: Acceptability of wearable inertial sensors, completeness of data, and day-to-day variability of everyday life motor activities in children and adolescents with neuromotor impairments
Source: Front Rehabil Sci. 2022 Dec 9;3:923328. doi: 10.3389/fresc.2022.923328 (PMC9788775; doi:10.3389/fresc.2022.923328)
Supplement: Supplementary file 1 [file Datasheet1.pdf]

Wearing time [h] (n = 43)

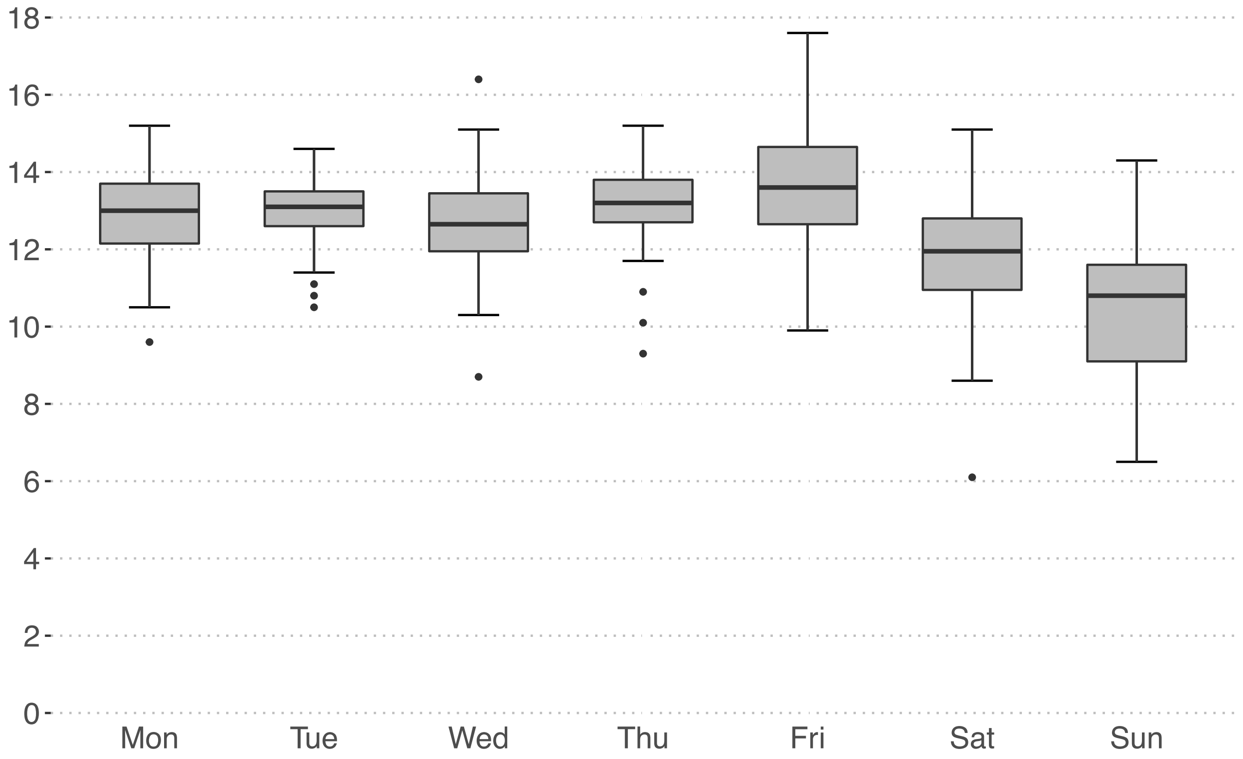

	Mon	Tue	Wed	Thu	Fri	Sat	Sun
Mon	12.9	1.00	0.94	0.96	0.15	0.00	0.00
Tue	+0.1	13.0	0.75	1.00	0.32	0.00	0.00
Wed	-0.3	-0.4	12.6	0.36	0.01	0.02	0.00
Thu	+0.2	+0.1	+0.5	13.1	0.66	0.00	0.00
Fri	+0.6	+0.5	+0.9	+0.4	13.5	0.00	0.00
Sat	-1.1	-1.2	-0.8	-1.4	-1.8	11.8	0.00
Sun	-2.5	-2.6	-2.3	-2.8	-3.2	-1.4	10.3

n = number of participants; upper triangle: tukey adjusted p-values; diagonal: estimated marginal means; lower triangle: estimated difference between weekdays

Functional activity counts (more affected hand) [counts] (n = 42)

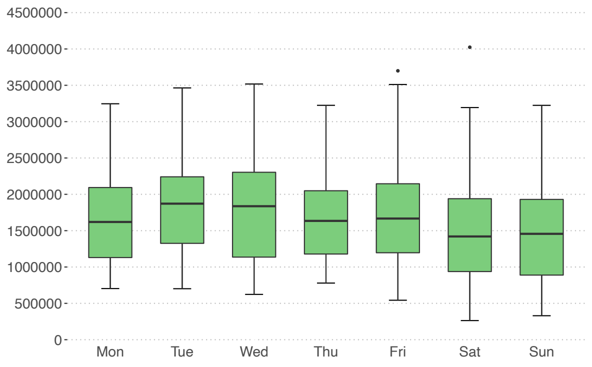

	Mon	Tue	Wed	Thu	Fri	Sat	Sun
Mon	1'689'003	0.96	1.00	1.00	0.72	0.65	0.06
Tue	+64'434	1'753'437	0.92	0.98	1.00	0.11	0.00
Wed	-11'295	-75'729	1'677'707	1.00	0.62	0.76	0.09
Thu	+8'578	-55'856	+19'873	1'697'581	0.75	0.50	0.03
Fri	+101'039	+36'605	+112'334	+92'461	1'790'042	0.02	0.00
Sat	-109'731	-174'165	-98'436	-118'309	-210'770	1'579'272	0.81
Sun	-205'349	-269'783	-194'054	-213'927	-306'388	-95'618	1'483'654

n = number of participants; upper triangle: tukey adjusted p-values; diagonal: estimated marginal means; lower triangle: estimated difference between weekdays

Functional activity counts (less affected hand) [counts] (n = 42)

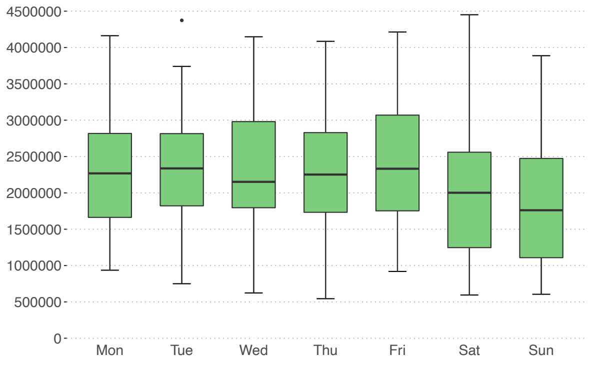

	Mon	Tue	Wed	Thu	Fri	Sat	Sun
Mon	2'312'892	0.99	0.99	1.00	0.85	0.17	0.00
Tue	+56'726	2'369'618	0.81	1.00	1.00	0.02	0.00
Wed	-55'031	-111'757	2'257'861	0.95	0.43	0.55	0.02
Thu	+23'997	-32'729	+79'028	2'336'888	0.94	0.05	0.00
Fri	+103'613	+46'887	+158'645	+79'617	2'416'505	0.00	0.00
Sat	-199'351	-256'077	-144'320	-223'348	-302'964	2'113'541	0.61
Sun	-342'069	-398'795	-287'037	-366'065	-445'682	-142'718	1'970'823

n = number of participants; upper triangle: tukey adjusted p-values; diagonal: estimated marginal means; lower triangle: estimated difference between weekdays

Functional activity counts (more affected/less affected hand) [%] (n = 42)

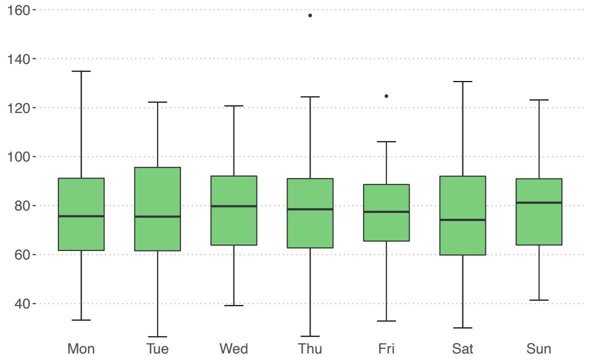

	Mon	Tue	Wed	Thu	Fri	Sat	Sun
Mon	75.7	0.93	1.00	1.00	1.00	1.00	0.93
Tue	+2.0	77.6	0.99	0.99	0.89	0.92	1.00
Wed	+0.7	-1.2	76.4	1.00	1.00	1.00	0.99
Thu	+0.6	-1.3	-0.1	76.3	1.00	1.00	0.98
Fri	-0.2	-2.1	-0.9	-0.8	75.5	1.00	0.89
Sat	+0.0	-1.9	-0.7	-0.6	+0.2	75.7	0.93
Sun	+2.1	+0.1	+1.4	+1.5	+2.2	+2.1	77.7

n = number of participants; upper triangle: tukey adjusted p-values; diagonal: estimated marginal means; lower triangle: estimated difference between weekdays

Duration in lying position [min] (n = 10)

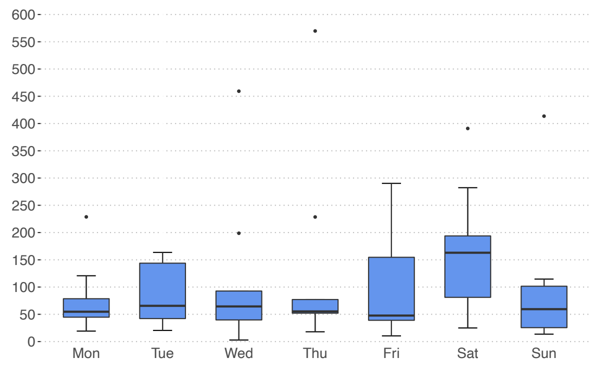

	Mon	Tue	Wed	Thu	Fri	Sat	Sun
Mon	87.2	1.00	0.98	0.87	1.00	0.49	0.94
Tue	+1.7	88.8	0.99	0.909	1.00	0.57	0.96
Wed	+33.1	+31.5	120.3	1.00	1.00	0.93	1.00
Thu	+50.0	+48.3	+16.8	137.1	1.00	0.99	1.00
Fri	+24.0	+22.3	-9.2	-26.0	111.2	0.88	1.00
Sat	+74.1	+72.5	+41.0	+24.1	+50.1	161.3	0.99
Sun	+45.8	+44.2	+12.7	-4.2	+21.8	-28.3	133.0

n = number of participants; upper triangle: tukey adjusted p-values; diagonal: estimated marginal means; lower triangle: estimated difference between weekdays

Duration in sitting position [min] (n = 10)

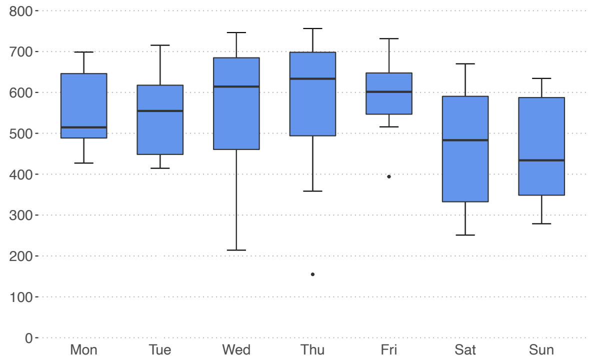

	Mon	Tue	Wed	Thu	Fri	Sat	Sun
Mon	539.0	1.00	1.00	1.00	0.99	0.70	0.56
Tue	+11.8	551.0	1.00	1.00	1.00	0.60	0.47
Wed	+7.0	-4.8	546.0	1.00	1.00	0.57	0.46
Thu	+14.3	+2.6	+7.4	553.0	1.00	0.46	0.38
Fri	+38.9	+27.1	+31.9	+24.6	578.0	0.28	0.20
Sat	-74.1	-85.9	-81.1	-88.5	-113.0	465.0	1.00
Sun	-96.6	-108.4	-103.6	-111.0	-135.5	-22.5	443.0

n = number of participants; upper triangle: tukey adjusted p-values; diagonal: estimated marginal means; lower triangle: estimated difference between weekdays

Duration in standing position [min] (n = 10)

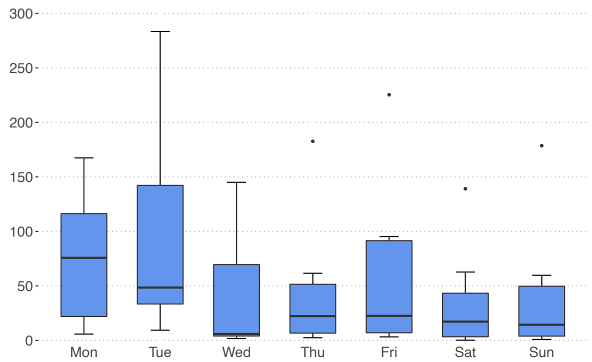

	Mon	Tue	Wed	Thu	Fri	Sat	Sun
Mon	68.7	0.92	0.44	0.62	0.98	0.24	0.29
Tue	+18.1	86.8	0.06	0.12	0.54	0.02	0.04
Wed	-30.5	-48.6	38.2	1.00	0.95	1.00	1.00
Thu	-26.7	-44.8	+3.8	42.0	0.98	1.00	0.99
Fri	-13.7	-31.8	+16.8	+13.0	55.0	0.80	0.79
Sat	-35.9	-53.9	-5.3	-9.1	-22.2	32.9	1.00
Sun	-38.8	-56.8	-8.3	-12.1	-25.1	-2.9	30.0

n = number of participants; upper triangle: tukey adjusted p-values; diagonal: estimated marginal means; lower triangle: estimated difference between weekdays

Active wheeling, distance [m] (n = 10)

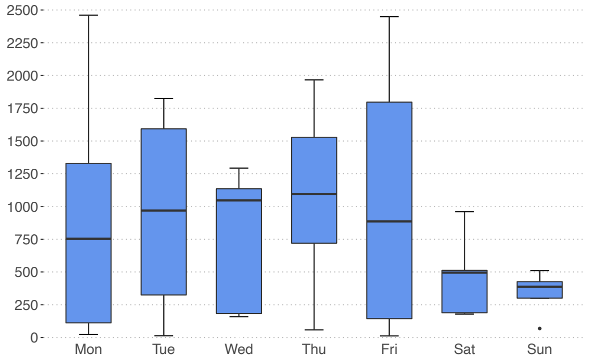

	Mon	Tue	Wed	Thu	Fri	Sat	Sun
Mon	833.0	1.00	0.96	1.00	0.97	0.44	0.58
Tue	+45.9	879.0	0.92	1.00	0.99	0.35	0.49
Wed	-284.1	-330.0	549.0	0.75	0.52	0.96	0.98
Thu	+142.5	+96.6	+426.6	975.0	1.00	0.14	0.28
Fri	+242.6	+196.7	+526.7	+100.2	1075.0	0.06	0.15
Sat	-562.7	-608.6	-278.6	-705.2	-805.3	270.0	1.00
Sun	-565.0	-610.9	-280.9	-707.5	-807.6	-2.3	268.0

n = number of participants; upper triangle: tukey adjusted p-values; diagonal: estimated marginal means; lower triangle: estimated difference between weekdays

Active wheeling, speed [m/s] (n = 10)

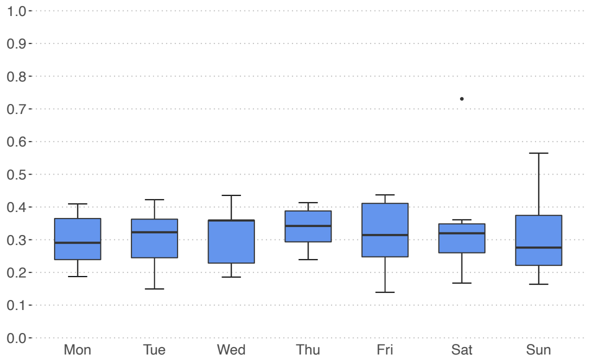

	Mon	Tue	Wed	Thu	Fri	Sat	Sun
Mon	0.29	1.00	1.00	0.97	1.00	0.96	1.00
Tue	0.00	0.29	1.00	0.98	1.00	0.98	1.00
Wed	0.00	0.00	0.29	0.99	1.00	0.98	1.00
Thu	+0.04	+0.04	+0.04	0.33	1.00	1.00	1.00
Fri	+0.03	+0.02	+0.02	-0.01	0.31	1.00	1.00
Sat	+0.05	+0.04	+0.04	0.00	+0.02	0.33	1.00
Sun	+0.01	+0.01	+0.01	-0.03	-0.02	-0.03	0.30

n = number of participants; upper triangle: tukey adjusted p-values; diagonal: estimated marginal means; lower triangle: estimated difference between weekdays

Active wheeling distance / total wheeling distance [%] (n = 10)

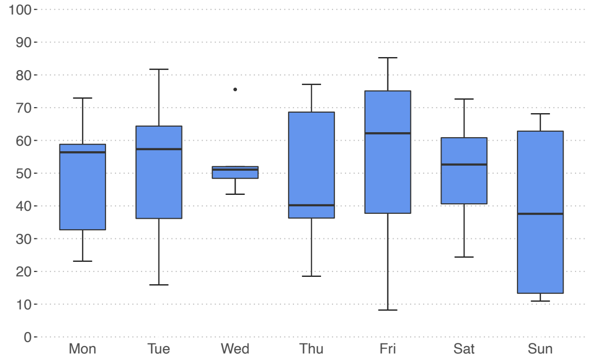

	Mon	Tue	Wed	Thu	Fri	Sat	Sun
Mon	46.9	1.00	1.00	1.00	1.00	1.00	0.67
Tue	+2.5	49.4	1.00	1.00	1.00	1.00	0.53
Wed	-0.0	-2.5	46.9	1.00	1.00	1.00	0.70
Thu	-2.8	-5.3	-2.8	44.1	0.97	1.00	0.80
Fri	+4.7	+2.3	+4.8	+7.5	51.6	0.99	0.36
Sat	-1.7	-4.2	-1.7	+1.1	-6.4	45.2	0.76
Sun	-17.6	-20.1	-17.6	-14.8	-22.3	-15.9	29.3

n = number of participants; upper triangle: tukey adjusted p-values; diagonal: estimated marginal means; lower triangle: estimated difference between weekdays

Walking duration [min] (n = 29)

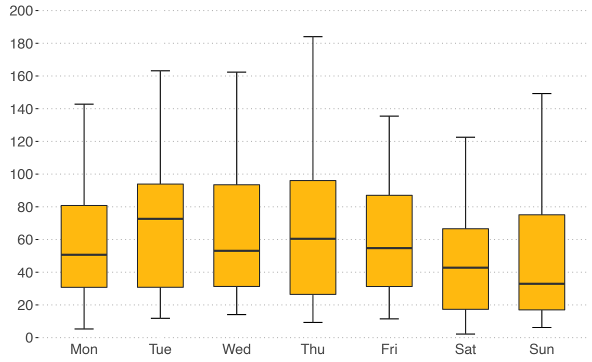

	Mon	Tue	Wed	Thu	Fri	Sat	Sun
Mon	60.4	0.29	1.00	0.94	1.00	0.69	0.45
Tue	+12.0	72.4	0.33	0.89	0.61	0.00	0.00
Wed	-0.1	-12.1	60.3	0.94	1.00	0.74	0.50
Thu	+5.7	-6.3	+5.8	66.1	1.00	0.10	0.04
Fri	+3.1	-8.9	+3.2	-2.6	63.5	0.29	0.14
Sat	-8.8	-20.8	-8.7	-14.5	-11.9	51.6	1.00
Sun	-11.0	-23.0	-10.9	-16.7	-14.1	-2.2	49.4

n = number of participants; upper triangle: tukey adjusted p-values; diagonal: estimated marginal means; lower triangle: estimated difference between weekdays

Assisted walking duration / total walking duration [%] (n = 9)

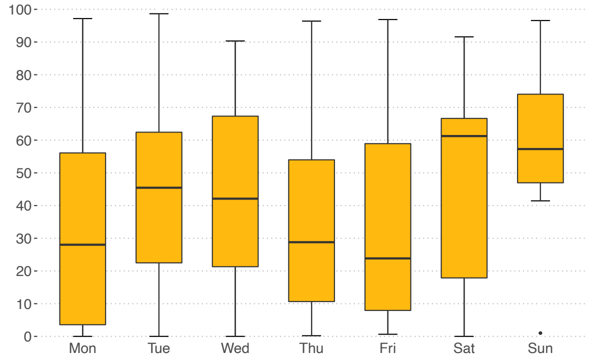

	Mon	Tue	Wed	Thu	Fri	Sat	Sun
Mon	63	0.74	0.99	1.00	0.95	0.93	1.00
Tue	-14.7	48.2	0.99	0.53	1.00	1.00	0.59
Wed	-7.5	+7.2	55.4	0.95	1.00	1.00	0.95
Thu	+2.6	+17.3	+10.1	65.5	0.83	0.78	1.00
Fri	-9.2	+5.5	-1.7	-11.8	53.7	1.00	0.85
Sat	-10.1	+4.7	-2.5	-12.6	-0.8	52.9	0.81
Sun	+3.3	+18.0	+10.8	+0.7	+12.6	+13.4	66.3

n = number of participants; upper triangle: tukey adjusted p-values; diagonal: estimated marginal means; lower triangle: estimated difference between weekdays

Walking distance [m] (n = 29)

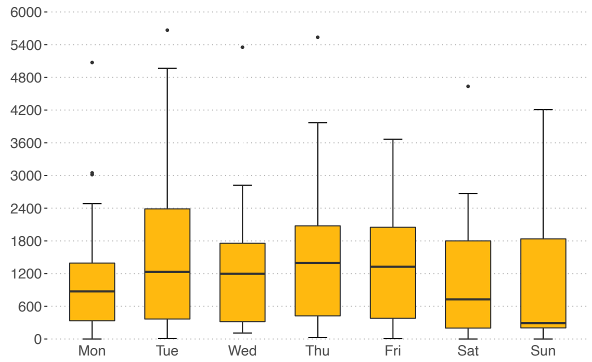

	Mon	Tue	Wed	Thu	Fri	Sat	Sun
Mon	1322	0.49	1.00	0.99	1.00	0.97	0.98
Tue	+343.6	1665	0.22	0.90	0.72	0.08	0.11
Wed	-106.0	-449.6	1216.0	0.85	0.97	1.00	1.00
Thu	+137.4	-206.1	+243.4	1459	1.00	0.62	0.69
Fri	+71.4	-272.2	+177.4	-66.0	1393	0.84	0.89
Sat	-168.2	-511.8	-62.2	-305.7	-239.6	1154	1.00
Sun	-152.7	-496.3	-46.7	-290.2	-224.1	+15.5	1169.0

n = number of participants; upper triangle: tukey adjusted p-values; diagonal: estimated marginal means; lower triangle: estimated difference between weekdays

Average walking speed [m/s] (n = 29)

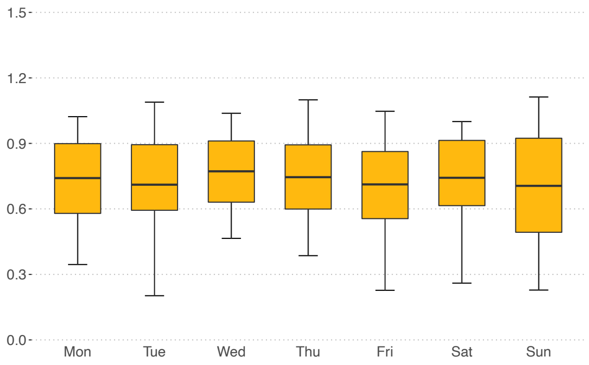

	Mon	Tue	Wed	Thu	Fri	Sat	Sun
Mon	0.74	0.95	1.00	1.00	0.94	0.99	0.43
Tue	-0.03	0.71	0.99	0.80	1.00	1.00	0.94
Wed	-0.01	+0.02	0.73	1.00	0.98	1.00	0.60
Thu	+0.01	+0.04	+0.01	0.75	0.75	0.90	0.19
Fri	-0.03	0.00	-0.02	-0.04	0.71	1.00	0.95
Sat	-0.02	0.00	-0.02	-0.03	+0.01	0.72	0.89
Sun	-0.05	-0.03	-0.05	-0.06	-0.03	-0.03	0.68

n = number of participants; upper triangle: tukey adjusted p-values; diagonal: estimated marginal means; lower triangle: estimated difference between weekdays

Going upstairs [m] (n = 26)

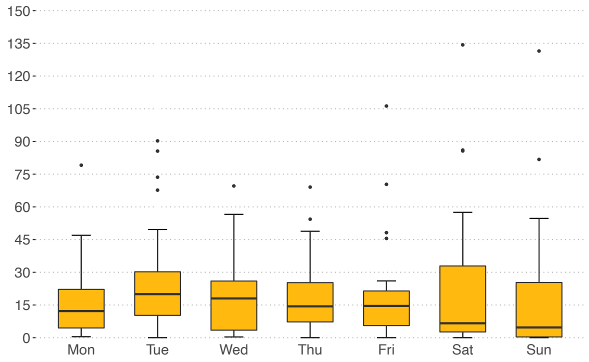

	Mon	Tue	Wed	Thu	Fri	Sat	Sun
Mon	18.4	0.77	1.00	1.00	1.00	0.92	1.00
Tue	+8.6	27.0	0.82	0.77	0.91	1.00	0.96
Wed	+0.1	-8.4	18.5	1.00	1.00	0.94	1.00
Thu	+0.4	-8.2	+0.3	18.8	1.00	0.92	1.00
Fri	+2.1	-6.5	+1.9	+1.7	20.4	0.98	1.00
Sat	+6.8	-1.8	+6.7	+6.4	+4.8	25.2	0.99
Sun	+2.7	-5.8	+2.6	+2.4	+0.7	-4.1	21.1

n = number of participants; upper triangle: tukey adjusted p-values; diagonal: estimated marginal means; lower triangle: estimated difference between weekdays

Going downstairs [m] (n = 26)

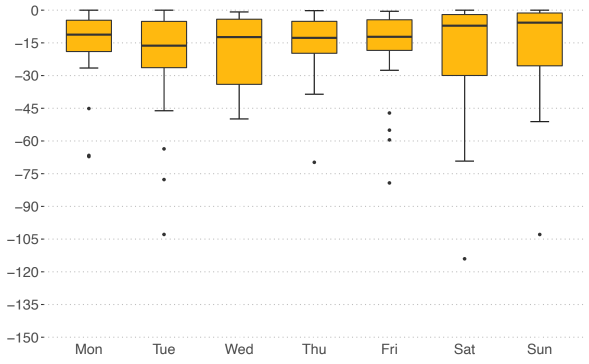

	Mon	Tue	Wed	Thu	Fri	Sat	Sun
Mon	-18.3	0.95	1.00	1.00	1.00	0.98	1.00
Tue	-5.1	-23.4	0.92	0.63	0.94	1.00	0.71
Wed	+0.8	+5.9	-17.5	1.00	1.00	0.97	1.00
Thu	+2.9	+8.0	+2.1	-15.3	1.00	0.78	1.00
Fri	+0.0	+5.1	-0.8	-2.9	-18.2	0.98	1.00
Sat	-4.1	+1.0	-4.9	-7.0	-4.1	-22.4	0.83
Sun	+2.9	+8.0	+2.1	-0.1	+2.8	+7.0	-15.4

n = number of participants; upper triangle: tukey adjusted p-values; diagonal: estimated marginal means; lower triangle: estimated difference between weekdays
